# Childhood trauma and negative memory bias as shared risk factors for psychopathology and comorbidity in a naturalistic psychiatric patient sample

**DOI:** 10.1002/brb3.693

**Published:** 2017-05-09

**Authors:** Janna N. Vrijsen, Camiel T. van Amen, Bauke Koekkoek, Iris van Oostrom, Aart H. Schene, Indira Tendolkar

**Affiliations:** ^1^ Department of PsychiatryRadboud University Medical CenterNijmegenThe Netherlands; ^2^ Pro Persona Mental Health CareDepression Expertise CenterNijmegenThe Netherlands; ^3^ Donders Institute for Brain Cognition and BehaviorCenter for NeuroscienceNijmegenThe Netherlands; ^4^ Research Group Social Psychiatry & Mental Health NursingHAN University of Applied SciencesNijmegenThe Netherlands; ^5^ Pro Persona Mental Health CareProCESWolfhezeThe Netherlands; ^6^ Department of PsychiatryAcademic Medical CenterAmsterdamThe Netherlands; ^7^ Department of Psychiatry and PsychotherapyUniversity Hospital EssenEssenGermany

**Keywords:** childhood trauma, cognitive bias, comorbidity, memory bias, psychiatry

## Abstract

**Background:**

Both childhood trauma and negative memory bias are associated with the onset and severity level of several psychiatric disorders, such as depression and anxiety disorders. Studies on these risk factors, however, generally use homogeneous noncomorbid samples. Hence, studies in naturalistic psychiatric samples are lacking. Moreover, we know little about the quantitative relationship between the frequency of traumatic childhood events, strength of memory bias and number of comorbid psychiatric disorders; the latter being an index of severity. The current study examined the association of childhood trauma and negative memory bias with psychopathology in a large naturalistic psychiatric patient sample.

**Methods:**

Frequency of traumatic childhood events (emotional neglect, psychological‐, physical‐ and sexual abuse) was assessed using a questionnaire in a sample of 252 adult psychiatric patients with no psychotic or bipolar‐I disorder and no cognitive disorder as main diagnosis. Patients were diagnosed for DSM‐IV Axis‐I and Axis‐II disorders using a structured clinical interview. This allowed for the assessment of comorbidity between disorders. Negative memory bias for verbal stimuli was measured using a computer task.

**Results:**

Linear regression models revealed that the frequency of childhood trauma as well as negative memory bias was positively associated with psychiatric comorbidity, separately and above and beyond each other (all *p* <* *.01).

**Conclusions:**

The results indicate that childhood trauma and negative memory bias may be of importance for a broader spectrum of psychiatric diagnoses, besides the frequently studied affective disorders. Importantly, frequently experiencing traumatic events during childhood increases the risk of comorbid psychiatric disorders.

## 1 Introduction

The lifetime prevalence of DSM‐IV (American Psychiatric Association, 1994) Axis‐I disorders is 46% (Kessler et al., 2005). Evidence‐based guidelines have been developed to address treatment for single Axis‐I and Axis‐II disorders, however, comorbidity of psychiatric disorders is highly frequent (Zimmerman, McGlinchey, Chelminski, & Young, 2008). For example, studies show that 17%–21% of psychiatric patients met the criteria for three or more psychiatric disorders (Andrews, Slade, & Issakidis, 2002; Kessler et al., 2005). Not only the co‐occurrence of DSM‐IV Axis‐I disorders, but also between Axis‐I and personality (Axis‐II) disorders is common (Links & Eynan, 2013). The high level of comorbidity suggests that similar pathogenic factors play a role in different psychiatric disorders. More knowledge about factors that influence the development, maintenance, recurrence, and co‐occurrence of psychiatric disorders can increase our understanding of psychiatric pathogenesis and might help in developing better diagnostic instruments, treatment options, and (relapse) prevention interventions.

Cognitive models have provided a solid framework to understand how the experience of adverse events, especially interpersonal trauma, during childhood contributes to the development psychopathology (Beck, 1967, 2008; Beck & Bredemeier, 2016). The experience of adverse or even traumatic events during childhood may result in dysfunctional basic assumptions about the self and the world. Information is processed in accordance with these assumptions, setting the stage for cognitive biases and increasing the risk for the development of psychiatric problems such as depression. The association between highly impactful negative childhood events, cognitive biases, and depression has been confirmed (Beck & Bredemeier, 2016; Scher, Ingram, & Segal, 2005), but childhood adversity is also an important and prevalent risk factor for psychiatric disorders other than depression, such as anxiety disorders, eating disorders, addiction, and sleep disorders (Chen et al., 2010; Hovens, Wiersma, Giltay, & van Oppen, 2010; Kessler, Davis, & Kendler, 1997; Spinhoven et al., 2010). The risk effect of impactful negative childhood events might thus be based on similar cognitive schemas across psychiatric disorders. Frequent occurrence of childhood trauma and a greater range of different types of childhood traumas increases the risk of the development of psychiatric disorders (Hovens et al., 2010). Hence it is important to take both the type of childhood trauma and the frequency of the events into account when studying psychopathology.

As said, the experience of childhood trauma may foster dysfunctional basic assumptions, which can result in negative cognitive biases (Beck, 2008). Negative cognitive bias is defined as preferential processing of negative material and has been found in different cognitive domains and in several psychiatric disorders (Arntz, Weertman, & Salet, 2011; Brooks, Prince, Stahl, Campbell, & Treasure, 2011; Mathews & MacLeod, 2005). Biased processing of emotional material in *memory* has frequently been studied and appears to be a robust cognitive emotional process, especially in depression (Gotlib & Joormann, 2010; Matt, Vazquez, & Campbell, 1992). Negative memory bias entails that negative material is more accessible in memory, resulting in more frequent and effective recall (Gotlib & Joormann, 2010).

Negative memory bias is a risk factor for the development, maintenance, and recurrence of depression (De Raedt & Koster, 2010; Gotlib & Joormann, 2010). In anxiety disorders, however, the evidence for memory bias is mixed (Mathews & MacLeod, 2005; Mitte, 2008). Furthermore, some recent studies found evidence for memory bias in disorders other than depression and anxiety, e.g., borderline personality disorder (Winter, Elzinga, & Schmahl, 2014), eating disorders (Nikendei et al., 2008), and schizophrenia (Peters, Hauschildt, Moritz, & Jelinek, 2013). The association between memory bias and psychiatric *comorbidity* is unclear at this point, but negative emotions are a key aspect of not only depression and anxiety, but of many psychiatric disorders (American Psychiatric Association, 1994). Therefore, we expect that the impact of childhood trauma on memory bias might in turn effects the level of comorbidity of psychiatric disorders (Zimmerman et al., 2008). We expect negative memory bias to be relevant for a broad spectrum of psychiatric disorders as well as the level of comorbidity between disorders. The latter might be driven by the specific comorbidity between depression and/or anxiety disorders with other disorders. This is however difficult to disentangle in a naturalistic psychiatric sample.

That childhood trauma and memory bias have mostly been studied in well‐defined depressed or anxious samples is in sharp contrast with psychiatric practice, where comorbidity between Axis‐I disorders, as well as between Axis‐I and Axis‐II disorders is high (Andrews et al., 2002; Links & Eynan, 2013; Winter et al., 2014). In order to gain the understanding of the global nature of these risk factors as well as their contribution to comorbidity, they need to be examined together in a large heterogenic naturalistic psychiatric patient sample. It is currently unknown if the frequency and diversity of childhood trauma has a cumulative effect on the development of psychiatric comorbidity. Not do we know if the strength of negative memory bias is related to the frequency and diversity of traumatic childhood events and the number of current psychiatric comorbidity as an index of the complexity and severity of the psychiatric problems. Hence, the aim of the current study was to explore the association of the frequency and diversity of childhood trauma and negative memory bias with a broad range of psychiatric disorders, as well as comorbidity between disorders. We expect more psychiatric diagnoses in patients with a higher frequency and diversity of childhood trauma, see studies showing a possible dose‐response relationship childhood trauma and psychiatric comorbidity (Hovens et al., 2010; Spinhoven et al., 2010). Furthermore, also negatively biased memory was expected to be associated with childhood trauma and the number of psychiatric diagnoses (Dozois & Dobson, 2001; Lemoult & Joormann, 2012). Moreover, in line with the cognitive models, the association between childhood trauma and comorbidity was expected to be mediated by negative memory bias. Taken together, the results of the study will provide an indication of influencing factors of psychiatric disorders and comorbidity in a naturalistic sample.

## 2 Method

### 2.1 Participants

This study is part of the MATCH Cohort Study (see Koekkoek, Manders, Tendolkar, Hutschemaekers, & Tiemens, 2016). The MATCH Cohort study is initiated by Research Group Social Psychiatry & Mental Health Nursing at the HAN University of Applied Sciences in Nijmegen, the Netherlands. A total of 285 psychiatric patients participated in the study. They were recruited at three large Dutch mental health institutes in the east of The Netherlands. Psychiatric patients were eligible if they met the criteria of a main psychiatric disorder according to the DSM‐IV, but had no psychotic or bipolar‐I disorder and no cognitive disorder as the main diagnosis. Patients with these main diagnoses were not invited because the psychiatric problems making the performance of the questionnaires and tasks too difficult. Participants were between 18 and 65 years of age and showed sufficient comprehension of the Dutch language. All eligible patients were invited for the study by means of an invitation letter. If a patient was willing to participate, a first appointment was made to obtain informed consent and collect the baseline data. Memory bias data of ten patients were missing. Subjects who did not recall any words were excluded from the analyses, because bias could not be measured (*n* = 10). The DSM‐IV diagnoses were not available for one participant; a further eleven participants did not fill out the NEMESIS‐childhood trauma questionnaire, and one participant did not complete the sociodemographic questionnaire, resulting in a final sample of 252 participants for analyses. Participants were rewarded a gift certificate for their participation. All procedures contributing to this work comply with the ethical standards of the relevant national and institutional committees on human experimentation and with the Helsinki Declaration of 1975, as revised in 2008. The study was approved by the local Medical Ethical Committee (“Commissie Mensgebonden Onderzoek regio Arnhem‐Nijmegen”).

### 2.2 Materials and apparatus

#### 2.2.1 Sociodemographics

Participant's gender, age in years and the highest level of education were assessed by means of an interview.

#### 2.2.2 Structured clinical interviews

The Mini International Neuropsychiatric Interview Plus (MINI Plus; Sheehan et al., 1997, 1998) is a validated structured diagnostic psychiatric interview. It was used by trained assessors to classify the DSM‐IV Axis‐I disorders within the following clusters: mood disorders, anxiety disorders, substance abuse, manic disorders, psychotic disorders, eating disorders, somatoform disorders, and ADHD. Because we were interested in comorbidity across disorders, we clustered specific disorders based on the main categories. For example, a patient with social phobia was classified as having “an anxiety disorder” without further specification of the type of anxiety disorder.

If a patient scored above the cut‐off on the Standardized Assessment of Personality‐Abbreviated Scale (Germans, Van Heck, & Hodiamont, 2012), the Structured Interview for DSM Personality Disorders (Pfohl, Blum, & Zimmerman, 1995) was used to classify the Axis‐II DSM‐IV personality disorders. The following personality disorders were assessed: schizoid, schizotypical, antisocial, borderline, histrionic, narcissistic, avoiding, dependent, and obsessive‐compulsive personality disorders.

#### 2.2.3 Childhood trauma

In line with Hovens et al. (2010) and Wiersma et al. (2009), childhood trauma was assessed retrospectively by a semi‐structured interview, previously used in the Netherlands Mental health Survey and Incidence Study (NEMESIS; De Graaf, Bijl, Ten Have, Beekman, & Vollebergh, 2004; De Graaf, Ten Have, & van Dorsselaer, 2010; Van Nierop et al., 2014). The NEMESIS‐childhood trauma questionnaire was used to identify stressful events endured by the subjects before the age of sixteen. Administration of the questionnaire took approximately 10 min and was done by a trained assessor. The questionnaire allows for the identifications of four meaningful types of traumatic childhood events within the family: emotional neglect, psychological abuse, physical abuse, and sexual abuse. The frequency of each type of childhood trauma was scored on a five‐point Likert scale, ranging from “0” (not) to “5” (very often). In accordance with the previous work on childhood trauma (Hovens et al., 2010), the outcomes of the frequencies of each type of traumatic childhood event were divided into three frequency scores: 0 represented “not,” 1 represented “once/sometimes” and 2 represented “regular/often/very often” occurrence of this type of traumatic childhood event. The “childhood trauma index” (Hovens et al., 2010) was calculated by taking the sum of the frequency scores of the four types of traumatic events and represented a combination of frequency and diversity of traumatic events. This index is highly correlated with the severity of psychopathology and the duration of depressive symptoms (see Hovens et al., 2010; Wiersma et al., 2009). This indicates that the childhood trauma index is a strong variable for examining the association between childhood trauma and psychopathology.

#### 2.2.4 Memory bias

The computerized Self Referent Encoding Task (SRET; Derry & Kuiper, 1981) was used to measure the memory bias. Memory bias is especially strong for self‐relevant information such as self‐descriptive adjectives (Matt et al., 1992; Symons & Johnson, 1997). The SRET is a reliable and stable tool for assessing the memory bias (Derry & Kuiper, 1981; Dobson & Shaw, 1987). During the encoding phase, 24 adjectives (twelve positively and twelve negatively valenced words) were sequentially presented in fixed random order on a computer screen with the restriction that not more than two words of the same valence would be presented sequentially. The participant was instructed to press the “j” (“ja” being Dutch for “yes”) key on the keyboard if the word described him/her and the “n” key if the word was not self‐descriptive. After a brief distraction, the participant was asked to type in as many words as he/she could remember from the previous task (i.e., the encoding phase) within 3 min. Spelling errors were permitted. The first two and the last two words were not included in the test results to reduce primacy and recency effects on the memory bias index. A negative memory bias index was calculated by dividing the number of endorsed and correctly recalled negative words by the total number of words endorsed and recalled (Gotlib et al., 2004).

### 2.3 Statistical analyses

Two variables representing the level of comorbidity were created: one indicating if comorbidity between psychiatric disorders was present (no disorder, 1 disorders diagnosed, 2 or more disorders diagnosed). The other comorbidity variable represented the total number of current psychiatric disorders. This variable was created by taking the sum of the main Axis‐I and personality (Axis‐II) disorders. Groups of overly treated patients without a current diagnosis are common in psychiatric care (Druss et al., 2007; Lawrence, Rasinski, Yoon, & Curlin, 2015; PLOS Medicine Editors, 2013), and were hence not excluded from the analyses in this naturalistic sample. Patients with and without traumatic childhood events, as well as patients with and without a current DSM‐IV diagnosis were compared on gender, age, and educational level using chi‐square analyses (categorical variables) and *t* tests. Age, gender, and education level were included as covariates, because they are associated with childhood events, psychopathology, and memory performance (Bjelland et al., 2008; Kendler, Kuhn, & Prescott, 2004). Direct linear associations between childhood trauma, negative memory bias, and comorbidity, as well as mediation by negative memory bias mediation were tested using the PROCESS tool for SPSS (Hayes, 2013). PROCESS bootstraps (1,000 samples) the indirect effect and outputs a 95% confidence interval. Note that if zero is not in the interval then the indirect effect is statistically significant at the .05 level.

## 3 Results

### 3.1 Sample descriptives

The sample descriptives can be found in Table 1. Patients with and patients without childhood trauma, as well as patients with and patients without a current psychiatric diagnosis did not differ on gender, age, or level of education: all *p* > .12. Because of the frequent co‐occurrence of DSM‐IV Axis‐I and Axis‐II personality disorder diagnoses in our naturalistic sample (i.e., 30.2% of participants), we did not examine the unique contribution of these types of disorders separately. Table 2 presents details on childhood trauma and psychiatric disorders in the sample.

**Table 1:** Sociodemographic characteristics of the sample. Absolute numbers and means (*SD*) with standard deviations, or percentages of gender, age, and education level

Variable	Absolute number (*N* = 252)	Means (*SD*) or percentage
Gender, female	179	71.0%
Age, years	—	38.2 (11.6)
Level of education^a^		
Lower education	9	3.6%
Lower vocational education	18	7.1%
Secondary general education	32	12.7%
Secondary vocational education	72	28.6%
High school	43	17.1%
Higher vocational education	49	19.4%
University	29	11.5%

a Highest educational level finished with a diploma.

**Table 2:** Mean (*SD*), range, and absolute numbers (percentages) of participants who experienced childhood trauma, and of patients diagnosed with one or two or more current DSM‐IV disorders specified for Axis‐I disorders, Axis‐II disorders, and any psychiatric disorders (Axis‐I and/or‐II)

Variable	Mean (*SD*)	Range	*n* (% of total)
Childhood trauma	3.1 (2.3)	0–8	201 (79.8%)
Negative memory bias	0.8 (0.4)	0–1	—
Current Axis‐I diagnosis	1.3 (1.1)	0–6	—
One	—	—	83 (32.9%)
Two or more	—	—	102 (41.7)
Current personality disorders diagnosis (Axis‐II)	0.7 (1.2)	0–6	—
One	—	—	37 (14.7)
Two or more	—	—	45 (18.3)
Current psychiatric diagnosis (Axis‐I and/or Axis‐II)	1.9 (1.9)	1–11	—
One	—	—	62 (24.6)
Two or more	—	—	133 (52.8)

### 3.2 Mediation model for the presence of comorbidity

**Figure 1:**
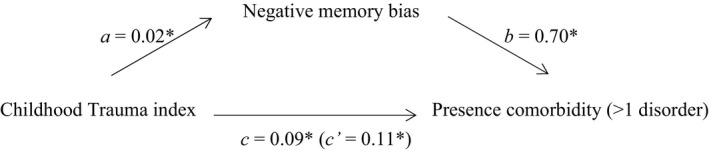
Standardized regression coefficients for the relationship between childhood trauma and the presence of comorbidity as mediated by negative memory bias. The standardized regression coefficient *c*′ between childhood trauma and comorbidity controlling for negative memory bias is in parentheses. *Significant at *p* < .05 level

A higher childhood trauma index score was significantly associated with the presence of comorbidity. The model is presented in Figure 1. More frequent and diverse childhood trauma was also significantly associated with more negatively biased memory, and negative memory bias was in turn significantly positively associated with the presence of comorbidity. Moreover, evidence for mediation was found: the relationship between childhood trauma and the presence of comorbidity was mediated by negative memory bias. The standardized indirect effect “ab” was .02. The ratio of the indirect effect to the direct effect was .19 (referred to as *P*_M_). The *P*_M_ value provides an indication of the effect size. The bootstrapped unstandardized indirect effect 95% confidence interval ranged from .006 to .03, indicating a significant indirect effect.

### 3.3 Diversity and frequency of childhood trauma

Post hoc analyses were conducted to explore whether diversity or frequency of childhood trauma drove the associations with negative memory bias and the presence of comorbidity. Two new childhood trauma variables were calculated: the diversity variable was the sum of the types of childhood trauma (range 0–4) an individual experienced and the frequency variable represented the total frequency rating across types of childhood trauma (range 0–20). Diversity of childhood trauma was significantly associated with both negative memory bias (*a *=* *0.04, *p *<* *.05) and the presence of comorbidity (*c *=* *0.16, *p *<* *.001). The latter association remained significant when negative memory bias was included in the model (*c*′ = .18, *p *<* *.001). As presented above and in Figure 1, negative memory bias and the presence of comorbidity were significantly associated. The standardized indirect effect “ab” was .03, *P*_M_ = .16, and the confidence interval ranged from .01 to .05, indicating mediation of the association between diversity of childhood trauma and the presence of comorbidity by negative memory bias. Evidence for mediation was also found when including the frequency of childhood trauma variable in the model. Frequency of trauma was significantly association with both negative memory bias (*a *=* *0.01, *p *<* *.05) and the presence of comorbidity (*c *=* *0.04, *p *<* *.001, *c′* = .05, *p *<* *.001). The standardized indirect effect “ab” was .01, *P*_M_ = .19, and the confidence interval ranged from .003 to .02. Comparing the coefficients and *P*_M_‐values (as indication of effect size) between the model using the childhood trauma diversity and model using the frequency variable, indicates that the mediation of negative memory bias may be stronger for frequency of childhood trauma.

### 3.4 Associations between childhood trauma, negative memory bias, and number of psychiatric disorders diagnosed

**Figure 2:**
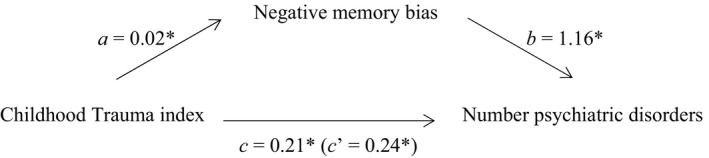
Standardized regression coefficients for the relationship between childhood trauma and number of psychiatric disorders diagnosed as mediated by negative memory bias. The standardized regression coefficient *c*′ between childhood trauma and number of diagnoses controlling for negative memory bias is in parentheses. *Significant at *p* < .05 level

More frequent/diverse childhood trauma was significantly associated with current higher total number of Axis‐I and Axis‐II psychiatric disorders diagnosed (i.e., indicating level of comorbidity). This model is presented in Figure 2. The first mediation model already showed that the childhood trauma index was significantly positively associated with negative memory bias. More negative memory bias was also significantly associated with a higher number of diagnoses. The relationship between childhood trauma and number of current psychiatric disorders diagnosed was mediated by negative memory bias with a standardized indirect effect “ab” of .03, and a *P*_M_ value of .11. The bootstrapped unstandardized indirect effect 95% confidence interval ranged from .01 to .06. Thus, the indirect effect was statistically significant.^1^

## 4 Discussion

In the current study, we substantiate and elaborate on first recent evidence on the relevance of memory bias in psychiatric disorders other than depression and anxiety (Nikendei et al., 2008; Winter et al., 2014). Our results reveal that both childhood trauma and negative memory bias are associated with comorbidity between psychiatric disorders. The current results once more highlight the detrimental effects of traumatic childhood events on psychiatric morbidity in adult life, as childhood trauma may have a cumulative effect on developing (comorbid) psychiatric disorders. Moreover, the high prevalence of childhood trauma in this naturalistic psychiatric patient sample (almost 80%) underscores its key role in psychological development. For comparison, in an independent sample 52% of depressed individuals experienced childhood trauma (measured with the same tool; De Graaf et al., 2004). Also the role of distorted cognitive processes in the association between childhood trauma and mental problems is indicated by the significant mediation effect.

Our results again indicate childhood trauma as risk factor for a broad spectrum of psychiatric disorders. Research has shown that depressed patients who experienced childhood trauma respond differently to treatment than patients without the experience of aggression or abuse during childhood (Nemeroff et al., 2003). They are more likely to have a recurrent or even chronic course of depression (Nanni, Uher, & Danese, 2012). It has been suggested that the neurobiological consequences of early trauma may lead to brain changes underlying cognitive factors causing and maintaining depression. Animal studies have demonstrated that chronic repeated stress evokes excitotoxicity changes in the hippocampus (McEwen, 2001) and smaller hippocampi has been linked to an increase in memory bias (Gerritsen et al., 2011), which in turn is thought to be a vulnerability factor for depression. Also, the impact of childhood trauma on changes in prefrontal and limbic brain structures can be found in various disorders (McCrory, De Brito, & Viding, 2012). This may explain the difference in course of psychiatric problems as well as in treatment responses.

Our findings in a naturalistic sample may have broader implications for diagnostics and clinical evaluations. The current findings support the idea that a higher frequency and diversity of traumatic childhood events may be a risk factor for a chronic course of multiple psychiatric disorders, as well as for diminishing responsiveness to treatment of disorders other than depression. Future studies will have to investigate whether such a frequency and diversity variable may be used as a semi‐quantitative vulnerability factor in clinical staging. Based on the positive association between the frequency and diversity of childhood trauma and negative memory bias, the latter may also serve as a valid biomarker in clinical staging. In the current study, memory bias was also associated with psychiatric comorbidity, even when patients with a current (comorbid) depression were excluded.^1^ Up to now, only a few studies investigated an association between negative memory bias and psychiatric disorders other than depression, or with psychiatric comorbidity (Dozois & Dobson, 2001; Lemoult & Joormann, 2012). These studies support the current results indicating that cognitive bias may be considered not only as a risk factor for the onset, maintenance, and recurrence of a disorder, but—if substantiated—also for comorbidity between psychiatric disorders (Brooks et al., 2011; De Raedt & Koster, 2010). Of course, this needs to be studies in much more detail before conclusions can be drawn.

Psychiatric entities—such as depression, bipolar disorder, ADHD, autism, and addiction—still prevail in clinical practice. Also in research, we still see a clear distinction between several psychiatric disorders; comorbidity between disorders is not often studied. However, the current results suggest that biased cognitive processing might play a role in a broad range of mental disorders, as well as in the development of comorbidity. Also it supports the current proposition that psychiatric disorders share underlying behavioral and neurobiological dimensions (Insel et al., 2010), one of which may be negative memory bias. Specific diagnoses based on symptomatology and neurobiological measures could give a more detailed image of psychiatric patients, which may result in improved personalized treatment.

In line with previous studies, we found evidence for high concurrence of DSM‐IV Axis‐I and Axis‐II disorders (Links & Eynan, 2013). This is not surprising given our choice for a naturalistic sample. Also, a group of 61 patients did not meet the DSM‐IV criteria for a current psychiatric disorder at the time of testing. These psychiatric patients generally show some psychiatric and/or psychosocial problems, and hence receive care largely outside the formal health care system. We expect this group to be less affected by psychiatric problems, however, all we can confirm is that they currently do not meet criteria for any psychiatric disorder. Groups of overly treated patients are rather common in psychiatric care (Druss et al., 2007; Lawrence et al., 2015; PLOS Medicine Editors, 2013).

The main limitation is that the current study does not have a prospective setup. Based on the model by Beck (2008) and Beck and Bredemeier (2016), we assumed that childhood trauma affects cognitive processing, which in turn increases the chance of developing a (comorbid) psychiatric disorder. Our data do not allow any conclusions about causality to be drawn. Moreover, psychopathology might have developed during childhood and we do not know if childhood trauma preceded the psychiatric problems. We do know from a meta‐analysis that the association between childhood trauma and psychopathology is present regardless of the age at which the events occurred (Chen et al., 2010). Another possible limitation is that recall of traumatic childhood events might have been biased due to present negative memory bias. However, recall of specific events is generally quite accurate (Brewin, Andrews, & Gotlib, 1993). To conclude, our result provide first evidence that childhood trauma and memory bias may be characteristic of, not only affective disorders, but a broader spectrum of psychiatric disorders as well as comorbidity between psychiatric disorders as indicator of severity of psychiatric problems. Importantly, the current study provides evidence for this association between risk factors in a naturalistic sample of psychiatric patients, and hence takes a step closer to the clinical practice where comorbidity is common.

## Conflict of Interest

There are no conflicts of interest to report.

## Note

1 Negative memory bias still mediated the association between childhood trauma and number of current psychiatric disorders diagnosed when excluding subjects with a current (comorbid) depression diagnosis (*n* = 77). All direct associations were significant at the *p < *.05 level. The standardized indirect effect “ab” was still .03 and the *P*_M_ value was .13. The bootstrapped unstandardized indirect effect 95% confidence interval ranged from .002, .06., indicating a statistically significant indirect effect.

## References

[brb3693-bib-0001] American Psychiatric Association (1994). Diagnostic and statistical manual of mental disorders (4th ed.). Washington, DC: Author.

[brb3693-bib-0002] Andrews, G. , Slade, T. , & Issakidis, C. (2002). Deconstructing current comorbidity: Data from the Australian National Survey of Mental Health and Well‐Being. British Journal of Psychiatry, 181, 306–314. 12356657 10.1192/bjp.181.4.306

[brb3693-bib-0003] Arntz, A. , Weertman, A. , & Salet, S. (2011). Interpretation bias in Cluster‐C and borderline personality disorders. Behaviour Research and Therapy, 49, 472–481. 21621746 10.1016/j.brat.2011.05.002

[brb3693-bib-0004] Beck, A. T. (1967). Depression: Clinical, experimental, and theoretical aspects (1st ed.). Philadelphia, PA: University of Pennsylvania Press.

[brb3693-bib-0005] Beck, A. T. (2008). The evolution of the cognitive model of depression and its neurobiological correlates. American Journal of Psychiatry, 165, 969–977. 18628348 10.1176/appi.ajp.2008.08050721

[brb3693-bib-0006] Beck, A. T. , & Bredemeier, K. (2016). A unified model of depression: Integrating clinical, cognitive, biological, and evolutionary perspectives. Clinical Psychological Science, 4(4), 596–619.

[brb3693-bib-0007] Bjelland, I. , Krokstad, S. , Mykletun, A. , Dahl, A. A. , Tell, G. S. , & Tambs, K. (2008). Does a higher educational level protect against anxiety and depression? The HUNT study. Social Science and Medicine, 66, 1334–1345. 18234406 10.1016/j.socscimed.2007.12.019

[brb3693-bib-0008] Brewin, C. R. , Andrews, B. , & Gotlib, I. H. (1993). Psychopathology and early experiences: A reappraisal of retrospective reports. Psychological Bulletin, 113, 82–98. 8426875 10.1037/0033-2909.113.1.82

[brb3693-bib-0009] Brooks, S. , Prince, A. , Stahl, D. , Campbell, I. C. , & Treasure, J. (2011). A systematic review and meta‐analysis of cognitive bias to food stimuli in people with disordered eating behaviour. Clinical Psychology Review, 31, 37–51. 21130935 10.1016/j.cpr.2010.09.006

[brb3693-bib-0010] Chen, L. P. , Murad, M. H. , Paras, M. L. , Colbenson, K. M. , Sattler, A. L. , Goranson, E. N. , … Zirakzadeh, A. (2010). Sexual abuse and lifetime diagnosis of psychiatric disorders: Systematic review and meta‐analysis. Mayo Clinic Proceedings, 85, 618–629. 20458101 10.4065/mcp.2009.0583 PMC2894717

[brb3693-bib-0011] De Graaf, R. , Bijl, R. V. , Ten Have, M. , Beekman, A. T. , & Vollebergh, W. A. (2004). Pathways to comorbidity: The transition of pure mood, anxiety and substance use disorders into comorbid conditions in a longitudinal population‐based study. Journal of Affective Disorders, 82, 461–467. 15555699 10.1016/j.jad.2004.03.001

[brb3693-bib-0012] De Graaf, R. , Ten Have, M , & van Dorsselaer, S. (2010). De psychische gezondheid van de Nederlandse bevolking. Nemesis‐2: Opzet en eerste resultaten. Trimbos, Utrecht, the Netherlands Retrieved from www.trimbos.nl/~/media/af0898%20nemesis%20ii.ashx

[brb3693-bib-0013] De Raedt, R. , & Koster, E. H. W. (2010). Understanding vulnerability for depression from a cognitive neuroscience perspective: A reappraisal of attentional factors and a new conceptual framework. Cognitive, Affective, & Behavioral Neuroscience, 10, 50–70. 10.3758/CABN.10.1.50 20233955

[brb3693-bib-0014] Derry, P. A. , & Kuiper, N. A. (1981). Schematic processing and self‐reference in clinical depression. Journal of Abnormal Psychology, 90, 286–297. 7264058 10.1037//0021-843x.90.4.286

[brb3693-bib-0015] Dobson, K. S. , & Shaw, B. F. (1987). Specificity and stability of self‐referent encoding in clinical depression. Journal of Abnormal Psychology, 96(1), 34–40. 3558947 10.1037//0021-843x.96.1.34

[brb3693-bib-0016] Dozois, D. J. A. , & Dobson, K. S. (2001). Information processing and cognitive organization in unipolar depression: Specificity and comorbidity issues. Journal of Abnormal Psychology, 110, 236–246. 11358018 10.1037//0021-843x.110.2.236

[brb3693-bib-0017] Druss, B. G. , Wang, P. S. , Sampson, N. A. , Olfson, M. , Pincus, H. A. , Wells, K. B. , & Kessler, R. C. (2007). Understanding mental health treatment in persons without mental diagnoses: Results from the National Comorbidity Survey Replication. Archives of General Psychiatry, 64, 1196–1203. 17909132 10.1001/archpsyc.64.10.1196 PMC2099263

[brb3693-bib-0018] Germans, S. , Van Heck, G. L. , & Hodiamont, P. P. (2012). Results of the search for personality disorder screening tools: Clinical implications. Journal of Clinical Psychiatry, 73, 165–173. 22401476 10.4088/JCP.11m07067

[brb3693-bib-0019] Gerritsen, L. , Tendolkar, I. , Franke, B. , Vasquez, A. A. , Kooijman, S. , Buitelaar, J. , … Rijpkema, M. (2011). BDNF Val66Met genotype modulates the effect of childhood adversity on subgenual anterior cingulate cortex volume in healthy subjects. Molecular Psychiatry, 17, 597–603. 21577214 10.1038/mp.2011.51

[brb3693-bib-0020] Gotlib, I. H. , & Joormann, J. (2010). Cognition and depression: Current status and future directions. Annual Review of Clinical Psychology, 6, 285–312. 10.1146/annurev.clinpsy.121208.131305 PMC2845726 20192795

[brb3693-bib-0021] Gotlib, I. H. , Kasch, K. L. , Traill, S. , Joormann, J. , Arnow, B. A. , & Johnson, S. L. (2004). Coherence and specificity of information‐processing biases in depression and social phobia. Journal of Abnormal Psychology, 113, 386–398. 15311984 10.1037/0021-843X.113.3.386

[brb3693-bib-0022] Hayes, A. F. (2013). Introduction to mediation, moderation and conditional process analysis (1st ed.). New York, NY: Guilford Press.

[brb3693-bib-0023] Hovens, J. G. , Wiersma, J. E. , Giltay, E. J. , & van Oppen, P. (2010). Childhood life events and childhood trauma in adult patients with depressive, anxiety and comorbid disorders vs. controls. Acta Psychiatrica Scandinavica, 122, 66–74. 19878136 10.1111/j.1600-0447.2009.01491.x

[brb3693-bib-0024] Insel, T. , Cuthbert, B. , Garvey, M. , Heinssen, R. , Pine, D. S. , Quinn, K. , … Wang, P. (2010). Research domain criteria (RDoC): Toward a new classification framework for research on mental disorders. American Journal of Psychiatry, 167, 748–751. 20595427 10.1176/appi.ajp.2010.09091379

[brb3693-bib-0025] Kendler, K. S. , Kuhn, J. , & Prescott, C. A. (2004). The interrelationship of neuroticism, sex and stressful life events in the prediction of episodes of major depression. American Journal of Psychiatry, 161, 631–636. 15056508 10.1176/appi.ajp.161.4.631

[brb3693-bib-0026] Kessler, R. C. , Berglund, P. B. , Demler, O. , Jin, R. , Merikangas, K. R. , & Walters, E. E. (2005). Lifetime prevalence and age‐of‐onset distributions of DSM‐IV disorders in the National Comorbidity Survey Replication. Archives of General Psychiatry, 62, 593–602. 15939837 10.1001/archpsyc.62.6.593

[brb3693-bib-0027] Kessler, R. C. , Davis, C. G. , & Kendler, K. S. (1997). Childhood adversity and adult psychiatric disorder in the US National Comorbidity Survey. Psychological Medicine, 27, 1101–1119. 9300515 10.1017/s0033291797005588

[brb3693-bib-0028] Koekkoek, B. , Manders, W. , Tendolkar, I. , Hutschemaekers, G. , & Tiemens, B. (2016). The MATCH cohort study in the Netherlands: Rationale, objectives, methods and baseline characteristics of patients with (long‐term) common mental disorders. International Journal of Methods in Psychiatric Research, 26(1). doi:10.1002/mpr.1512 [Epub ahead of print]. 10.1002/mpr.1512 PMC6877124 27307353

[brb3693-bib-0029] Lawrence, R. E. , Rasinski, K. A. , Yoon, J. D. , & Curlin, F. A. (2015). Psychiatrists’ and primary care physicians’ beliefs about overtreatment of depression and anxiety. The Journal of Nervous and Mental Disease, 203, 120–125. 25594787 10.1097/NMD.0000000000000247

[brb3693-bib-0030] Lemoult, J. , & Joormann, J. (2012). Attention and memory biases in social anxiety disorder: The role of comorbid depression. Cognitive Therapy and Research, 36, 47–57. 23087492 10.1007/s10608-010-9322-2 PMC3475322

[brb3693-bib-0031] Links, P. S. , & Eynan, R. (2013). The relationship between personality disorders and Axis‐I psychopathology: Deconstructing comorbidity. Annual Review of Clinical Psychology, 9, 529–554. 10.1146/annurev-clinpsy-050212-185624 23157449

[brb3693-bib-0032] Mathews, A. , & MacLeod, C. (2005). Cognitive vulnerability to emotional disorders. Annual Review of Clinical Psychology, 1, 167–195. 10.1146/annurev.clinpsy.1.102803.143916 17716086

[brb3693-bib-0033] Matt, G. E. , Vazquez, C. , & Campbell, W. K. (1992). Mood‐congruent recall of affectively toned stimuli: A meta‐analytic review. Clinical Psychology Review, 12, 227–255.

[brb3693-bib-0034] McCrory, E. , De Brito, S. A. , & Viding, E. (2012). The link between child abuse and psychopathology: A review of neurobiological and genetic research. Journal of the Royal Society of Medicine, 105, 151–156. 22532655 10.1258/jrsm.2011.110222 PMC3343716

[brb3693-bib-0035] McEwen, B. S. (2001). Plasticity of the hippocampus: Adaptation to chronic stress and allostatic load. Annals of the New York Academy of Sciences, 933, 265–277. 12000027 10.1111/j.1749-6632.2001.tb05830.x

[brb3693-bib-0036] Mitte, K. (2008). Memory bias for threatening information in anxiety and anxiety disorders: A meta‐analytic review. Psychological Bulletin, 134, 886–911. 18954160 10.1037/a0013343

[brb3693-bib-0037] Nanni, V. , Uher, R. , & Danese, A. (2012). Childhood maltreatment predicts unfavorable course of illness and treatment outcome in depression: A meta‐analysis. American Journal of Psychiatry, 169, 141–151. 22420036 10.1176/appi.ajp.2011.11020335

[brb3693-bib-0038] Nemeroff, C. B. , Heim, C. M. , Thase, M. E. , Klein, D. N. , Rush, A. J. , Schatzberg, A. F. , … Keller, M. B. (2003). Differential responses to psychotherapy versus pharmacotherapy in patients with chronic forms of major depression and childhood trauma. Proceedings of the National Academy of Sciences of the United States of America, 100, 14293–14296. 14615578 10.1073/pnas.2336126100 PMC283585

[brb3693-bib-0039] Nikendei, C. , Weisbrod, M. , Schild, S. , Bender, S. , Walther, S. , Herzog, W. , … Friederich, H. C. (2008). Anorexia nervosa: Selective processing of food‐related word and pictorial stimuli in recognition and free recall tests. International Journal of Eating Disorders, 41, 439–447. 18348282 10.1002/eat.20518

[brb3693-bib-0040] Peters, M. J. V. , Hauschildt, M. , Moritz, S. , & Jelinek, L. (2013). Impact of emotionality on memory and meta‐memory in schizophrenia using video sequences. Journal of Behavior Therapy and Experimental Psychiatry, 44, 77–83. 22925714 10.1016/j.jbtep.2012.07.003

[brb3693-bib-0041] Pfohl, B. , Blum, N. , & Zimmerman, M. (1995). Structured interview for DSM‐IV personality disorders (SIDP‐IV) (1st ed.). Iowa City, IA: American Psychiatry Press, Inc..

[brb3693-bib-0042] PLOS Medicine Editors (2013). The paradox of mental health: Over‐treatment and under‐recognition. Plos Medicine, 10, e1001456. doi:10.1371/journal.pmed.1001456 23723743 10.1371/journal.pmed.1001456 PMC3665855

[brb3693-bib-0043] Scher, C. , Ingram, R. , & Segal, Z. (2005). Cognitive reactivity and vulnerability: Empirical evaluation of construct activation and cognitive diatheses in unipolar depression. Clinical Psychology Review, 25, 417–510. 10.1016/j.cpr.2005.01.005 15914266

[brb3693-bib-0044] Sheehan, D. V. , Lecrubier, Y. , Harnett‐Sheehan, K. , Janavs, J. , Weiller, E. , Keskiner, A. , … Dunbar, G. C. (1997). The validity of the Mini International europsychiatric Interview (M.I.N.I.) according to the SCID‐P and its reliability. European Psychiatry, 12, 232–241.

[brb3693-bib-0045] Sheehan, D. V. , Lecrubier, Y. , Sheehan, K. H. , Amorim, P. , Janavas, J. , Weiller, E. , … Dunbar, G. C. (1998). The Mini‐International Neuropsychiatric Interview (M.I.N.I.): The development and validation of a structured diagnostic psychiatric interview for dsm‐iv and icd‐10. Journal of Clinical Psychiatry, 59, 22–33. 9881538

[brb3693-bib-0046] Spinhoven, P. , Elzinga, B. M. , Hovens, J. G. , Roelofs, K. , Zitman, F. G. , van Oppen, P. , & Penninx, B. W. (2010). The specificity of childhood adversities and negative life events across the lifespan to anxiety and depressive disorders. Journal of Affective Disorders, 126, 103–112. 20304501 10.1016/j.jad.2010.02.132

[brb3693-bib-0047] Symons, C. S. , & Johnson, B. T. (1997). The self‐reference effect in memory: A meta‐analysis. Psychological Bulletin, 121(3), 371–394. 9136641 10.1037/0033-2909.121.3.371

[brb3693-bib-0048] Van Nierop, M. , Van Os, J. , Gunther, N. , Van Zelst, C. , de Graaf, R. , ten Have, M. , … van Winkel, R. (2014). Does social defeat mediate the association between childhood trauma and psychosis? Evidence from the NEMESIS‐2 Study. Acta Psychiatrica Scandinavica, 129, 467–476. 24571736 10.1111/acps.12212

[brb3693-bib-0049] Wiersma, J. E. , Hovens, J. G. , van Oppen, P. , Giltay, E. J. , van Schaik, D. J. , Beekman, A. T. , & Penninx, B. W. (2009). The importance of childhood trauma and childhood life events for chronicity of depression in adults. Journal of Clinical Psychiatry, 70(7), 983–989. 19653975 10.4088/jcp.08m04521

[brb3693-bib-0050] Winter, D. , Elzinga, B. M. , & Schmahl, C. (2014). Emotions and memory in borderline personality disorder. Psychopathology, 47, 71–85. 24355827 10.1159/000356360

[brb3693-bib-0051] Zimmerman, M. , McGlinchey, J. B. , Chelminski, I. , & Young, D. (2008). Diagnostic co‐morbidity in 2300 psychiatric out‐patients presenting for treatment evaluated with a semi‐structured diagnostic interview. Psychological Medicine, 38, 199. 17949515 10.1017/S0033291707001717

