# Childhood trauma and negative memory bias as shared risk factors for psychopathology and comorbidity in a naturalistic psychiatric patient sample

**DOI:** 10.1002/brb3.1181

**Published:** 2018-12-26

**Authors:** Janna N. Vrijsen, Camiel T. van Amen, Bauke Koekkoek, Iris van Oostrom, Aart H. Schene, Indira Tendolkar

The authors would like to apologize for the errors in the original paper of Vrijsen et al. (2017). Due to mislabeling of one of the childhood trauma variables, the childhood trauma index scores of some participants were incorrect. Upon correction of the error, the pattern of results remained the same, resulting in the same conclusions. The description of the labeling of the childhood trauma variables on page 3 should be as follows: “The frequency per type of childhood trauma was scored on a five‐point Likert scale: 0 = not, 1 = once, 2 = sometimes, 3 = regularly, 4 = often, 5 = very often. Only ‘emotional neglect’ was scored on a four‐point Likert scale (0‐4, without the answer option ‘once’).” This also altered the range of the childhood trauma frequency score, resulting in a correction on page 5, “the diversity variable was the sum of the types of childhood trauma (possible range 0‐4) an individual experienced and the frequency variable represented the total frequency rating across types of childhood trauma (possible range 0‐19).”

In the “Mediation model for presence of comorbidity” section on page 4, the results now read *“*The standardized indirect effect ‘ab’ was 0.01. The ratio of the indirect effect to the direct effect was 0.17 (referred to as *P*_M_). The *P*_M_ value provides an indication of the effect size. The bootstrapped unstandardized indirect effect 95% confidence interval ranged from 0.005 to 0.03, indicating a significant indirect effect.”

In the “Associations between childhood trauma, negative memory bias, and number of psychiatric disorders diagnosed” section on page 5, the results now read: “The relationship between childhood trauma and number of current psychiatric disorders diagnosed was mediated by negative memory bias with a standardized indirect effect ‘ab’ of 0.02, and a *P*_M_ value of 0.12. The bootstrapped unstandardized indirect effect 95% confidence interval ranged from 0.01 to 0.06.” Furthermore, the corrected footnote on page 5 reads: “The standardized indirect effect ‘ab’ was still 0.02 and the *P*_M_ value was 0.12.”

In Table 2, the variable of “Negative memory bias” mean should be “0.3” instead of “0.8”.

Figures 1, 2 have also been corrected accordingly, below are the updated figures.

**Figure 1:**
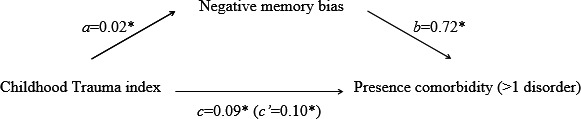


**Figure 2:**
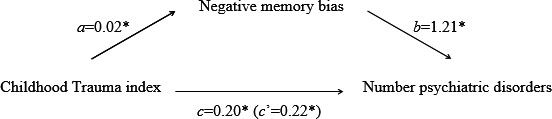


The author names of the reference Hoven et al. (2010) have been updated to the below:

- Hovens, J. G., Wiersma, J. E., Giltay, E. J., van Oppen, P., Spinhoven, P., Penninx, B. W., & Zitman, F.G. (2010). Childhood life events and childhood trauma in adult patients with depressive, anxiety, and comorbid disorders vs. controls. *Acta Psychiatrica Scandinavica*,* 122*, 66–74.
